# Increased risk of dementia in patients hospitalized with acute kidney injury: A nationwide population-based cohort study

**DOI:** 10.1371/journal.pone.0171671

**Published:** 2017-02-13

**Authors:** Hsin-Hsi Tsai, Ruoh-Fang Yen, Cheng-Li Lin, Chia-Hung Kao

**Affiliations:** 1 Department of Neurology, National Taiwan University Hospital, Taipei, Taiwan; 2 Department of Nuclear Medicine, National Taiwan University Hospital, Taipei, Taiwan; 3 Department of Radiology, National Taiwan University College of Medicine, Taipei, Taiwan; 4 Management Office for Health Data, China Medical University Hospital, Taichung, Taiwan; 5 College of Medicine, China Medical University, Taichung, Taiwan; 6 Graduate Institute of Clinical Medical Science and School of Medicine, College of Medicine, China Medical University, Taichung, Taiwan; 7 Department of Nuclear Medicine and PET Center, China Medical University Hospital, Taichung, Taiwan; 8 Department of Bioinformatics and Medical Engineering, Asia University, Taichung, Taiwan; University of Sao Paulo Medical School, BRAZIL

## Abstract

**Purpose:**

To determine whether acute kidney injury (AKI) is a risk factor for dementia.

**Methods:**

This nationwide population-based cohort study was based on data from the Taiwan National Health Insurance Research Database for 2000–2011. The incidence and relative risk of dementia were assessed in 207788 patients hospitalized for AKI. The comparison control was selected using the propensity score based on age, sex, index year and comorbidities.

**Results:**

During the 12-year follow-up, patients with AKI had a significantly higher incidence for developing dementia than did the controls (8.84 vs 5.75 per 1000 person-y). A 1.88-fold increased risk of dementia (95% confidence interval, 1.76–2.01) was observed after adjustment for age, sex, and several comorbidities (diabetes, hypertension, hyperlipidemia, head injury, depression, stroke, chronic obstructive pulmonary disease, coronary artery disease, congestive heart failure, atrial fibrillation, cancer, liver disease, chronic infection/inflammation, autoimmune disease, malnutrition).

**Conclusions:**

We found that patients with AKI exhibited a significantly increased risk of developing dementia. This study provides evidence on the association between AKI and long-term adverse outcomes. Additional clinical studies investigating the related pathways are warranted.

## Introduction

Dementia is a neurodegenerative syndrome characterized by progressive deterioration in cognitive ability and capacity for independent living [1]. Its prevalence increases exponentially with age. Because of increased life expectancy, the number of dementia cases is expected to rise substantially [2], which poses a considerable challenge for public health and social policy [3].

Chronic renal impairment, or end-stage renal disease (ESRD), is associated with accelerated cardiovascular events, cognitive impairment, and dementia [4–7]. Frequent vascular comorbidities, such as hypertension, diabetes mellitus, and dyslipidemia, also play a role in dementia development [8]. However, no study has investigated the relationship between acute kidney injury (AKI) and dementia, probably because of its reversible nature. AKI refers to a sudden decline in renal function that causes disturbances in fluid, electrolyte, and acid-base balance. AKI is a common complication in hospitalized or critically ill patients [9, 10], and its risk increases with age and pre-existing comorbidities [11, 12]. The long-term impact of AKI remains controversial, and most of the observational studies focused only on future dialysis and survival [13]. In addition to unfavorable long-term renal outcome, previous studies have discovered that patients who recover from AKI have a predisposition to cardiovascular and cerebrovascular events [9, 13–16]. Whether the episode of AKI may further contribute to the cognitive decline raises an important and interesting question.

In the current nationwide cohort study, we hypothesized that hospitalized patients surviving AKI would have higher probability of developing dementia in the long term. We selected 207788 patients with AKI and 207788 patients without AKI from the Taiwan National Health Insurance Research Database (NHIRD) to investigate the association between AKI and dementia.

## Methods

### Data source

The National Health Insurance (NHI) program is a nationwide single-payer health insurance system established by the Taiwanese Bureau of National Health Insurance in March 1995. It is a compulsory insurance for Taiwanese citizens, and the coverage rate is over 99%. The NHIRD comprises claims data of patients enrolled in the NHI program. The identities of beneficiaries in the database are recoded before being released to researchers. In the NHIRD, disease identification is based on the International Classification of Diseases, 9th Revision, Clinical Modification (ICD-9-CM), which was coded by a qualified clinician according to the clinical assessment, including disease presentation, physical findings and laboratory tests. Medical reimbursement specialists and peer review scrutinized all the insurance claims based on the standard diagnosed criteria.

### Ethics statement

The NHIRD encrypts patient personal information to protect privacy and provides researchers with anonymous identification numbers associated with relevant claims information, including sex, date of birth, medical services received, and prescriptions. Therefore, patient consent is not required to access the NHIRD. This study was approved to fulfill the condition for exemption by the Institutional Review Board (IRB) of China Medical University (CMUH104-REC2-115-CR1). The IRB also specifically waived the consent requirement.

### Sample participants

Patients with newly diagnosed AKI (ICD-9-CM Code 584) from 2000 to 2011 were identified (*n* = 234314) from the inpatient claims. The date of admission with initial AKI diagnosis was set as the index date. We excluded patients who had received a diagnosis for chronic kidney disease (CKD; ICD-9-CM Codes 580–589), ESRD (ICD-9-CM Code 585), or dementia (ICD-9-CM Codes 290, 294.1, 331.0) at the baseline (*n* = 10117). Those who were aged under 20 years (*n* = 3502), who had incomplete demographic information (*n* = 2), or unable to find a match control (*n* = 12905) were also excluded. One control without the diagnosis of AKI was matched with each AKI case. The exclusion criteria for controls were identical as case cohorts. For each patient with AKI, the corresponding control was selected based on the propensity score with a nearest neighbor algorithm and without replacement. The propensity score-matching criteria were index-year, age, sex, and comorbidities (diabetes, hypertension, hyperlipidemia, head injury, depression, stroke, chronic obstructive pulmonary disease (COPD), coronary artery disease (CAD), congestive heart failure (CHF), atrial fibrillation (AF), cancer, liver disease, chronic infection/inflammation, autoimmune disease, malnutrition). A total of 207788 patients with AKI and 207788 controls without AKI were included in this study.

### Data availability statement

All data and related metadata were deposited in an appropriate public repository in the National Health Research Institutes (NHRI). The NHRI is a nonprofit foundation established by the government. Only citizens of the Republic of China (Taiwan) who fulfill the requirements of conducting research projects are eligible to apply for the NHIRD. The use of NHIRD is limited to research purposes only. Applicants must follow the Computer-Processed Personal Data Protection Law (http://www.winklerpartners.com/?p=987) and related regulations of National Health Insurance Administration and NHRI. The applicant and his/her supervisor must sign an agreement upon application submission. All applications are reviewed for approval of data release.

### Outcome and comorbidities

All patients were followed from the index date until the date of dementia development (ICD-9-CM Codes 290, 294.1, 331.0). Those who did not develop dementia were censored at death, withdrawal from the insurance system, or the end of 2011 (December 31, 2011), whichever came first. Patients who develop CKD (ICD-9-CM Codes 580–589) after AKI were also identified.

We assessed the confounding factors of age, sex, and dementia-associated comorbidities. The dementia-associated comorbidities were diabetes (ICD-9-CM Code 250), hypertension (ICD-9-CM Codes 401–405), hyperlipidemia (ICD-9-CM Code 272), head injury (ICD-9-CM Codes 310.2, 800, 801, 803, 804, 850, 851, 853, 854), depression (ICD-9-CM Codes 296.2, 296.3, 300.4, 311), stroke (ICD-9-CM Codes 430–438), COPD (ICD-9-CM Codes 490–492,494, 496), CAD (ICD-9-CM Codes 410–414), CHF (ICD-9-CM Code 428), AF (ICD-9-CM Codes 427.31 and 427.3), cancer (ICD-9-CM Codes 140–208), liver disease (ICD-9-CM Codes 571, 070), chronic infection/inflammation (ICD-9-CM Codes 042–044, 010–018, 090–099), autoimmune disease (ICD-9-CM Codes 279), and malnutrition (ICD-9-CM Codes 260–269).

### Statistical analysis

Between the AKI and non-AKI cohorts, we calculated the frequency and percentage for the categorical variables and the mean and SD for the continuous variables. The distribution differences between the 2 cohorts were assessed using standardized mean differences. A standardized mean difference of ≤0.1 indicates a negligible difference between the two cohorts.

The cumulative incidence curves for dementia for both cohorts were plotted using the Kaplan–Meier method. The log rank test was applied to test the curves. The incidence density of subsequent dementia for each cohort was estimated according to the number of dementia events divided by the total follow-up (per 1000 person-y). Cox proportional hazards models stratifying on the matched pairs were performed to estimate the hazard ratio (HR) and 95% confidence intervals (CI) of developing dementia associated with AKI, compared with non-AKI cohort. We executed all data analyses by using SAS Version 9.3 (SAS Institute, Inc., Cary, NC, USA). The level of significance was set to *P* < .05 and the tests were 2-tailed.

## Results

Table 1 shows the demographic data and comorbidities for the patients with AKI (*n* = 207788) and the controls (*n* = 207788). The mean ages of the AKI and control cohorts were 68.13 (±16.08 y) and 68.57 (±14.97 y), respectively. Approximately 60.80% of the patients were male in AKI cohort. Comorbidities, including diabetes (35.81% vs 36.14%), hypertension (48.57% vs 49.59%), hyperlipidemia (10.64% vs 10.89%), head injury (7.30% vs 7.72%), depression (2.60% vs 2.71%), stroke (22.72% vs 22.74%), COPD (15.07% vs 14.92%), CAD (26.62% vs 21.86%), CHF (13.94% vs 14.20%), AF (6.83% vs 6.77%), cancer (17.86% vs 18.24%), liver disease (20.46% vs 21.01%), chronic infection/inflammation (5.08% vs 4.95%), autoimmune disease (0.67% vs 0.67%), and malnutrition (1.59% vs 1.56%) were comparable and did not significantly differ between the two cohorts.

**Table 1:** Distribution of age, sex, and comorbidity between the patients with AKI and the comparison cohort.

	Acute kidney injury				
	Yes (N = 207788)		No (N = 207788)		
	n	%	n	%	Standardized mean differences^§^
**Age, year**					
≦49	32154	15.47	26296	12.66	0.08
50–64	43137	20.76	44695	21.51	0.02
65–79	78573	37.81	87690	42.20	0.09
≥ 80	53924	25.95	49107	23.63	0.05
Mean (SD)	68.13	16.08	68.57	14.97	0.03
**Sex**					
Female	81460	39.20	83011	39.95	0.02
Male	126328	60.80	124777	60.05	0.02
**Comorbidity**					
Diabetes	74412	35.81	75089	36.14	0.01
Hypertension	100930	48.57	103043	49.59	0.02
Hyperlipidemia	22104	10.64	22621	10.89	0.01
Head injury	15174	7.30	16035	7.72	0.02
Depression	5411	2.60	5624	2.71	0.01
Stroke	47211	22.72	47257	22.74	0.001
COPD	31305	15.07	31011	14.92	0.00
CAD	44929	26.62	45431	21.86	0.01
CHF	28976	13.94	29501	14.20	0.01
AF	14183	6.83	14064	6.77	0.00
Cancer	37110	17.86	37891	18.24	0.01
Liver disease	42522	20.46	43661	21.01	0.01
Chronic infection/inflammation	10553	5.08	10284	4.95	0.01
Autoimmune disease	1393	0.67	1400	0.67	0.00
Malnutrition	3310	1.59	3247	1.56	0.00

^§^A standardized mean difference of ≤0.10 indicates a negligible difference between the two cohorts.

**Fig 1:**
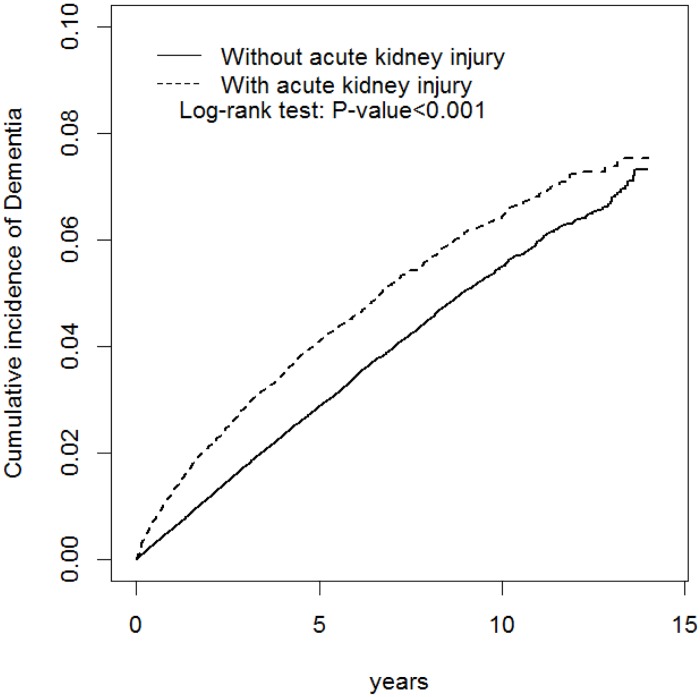
Cumulative incidence of dementia compared between the AKI patients and controls.

During the 12-year follow-up, patients with AKI had a significantly higher overall incidence of dementia than did the controls (8.84 vs 5.75 per 1000 person-y), with a HR of 1.88 (95% CI = 1.76–2.01). After stratification by age, sex, and comorbidity status, the risk of dementia in the patients with AKI remained statistically higher than in the controls (Table 2). Fig 1 demonstrates the Kaplan–Meier cumulative incidence curves of dementia in the AKI and control groups (log-rank test *P* < .001). In patients with AKI (*n* = 207788), 14658 of them later developed CKD (7.05%). The risk of dementia development was still significantly higher in AKI patients without a later CKD diagnosis (n = 193130, aHR = 1.76, 95% CI = 1.68–1.85).

**Table 2:** Incidence and hazard ratio of dementia in the patients with AKI and the comparison cohort.

	Acute kidney injury						
	Yes			No			
Outcome	Event	PY	Rate^#^	Event	PY	Rate^#^	HR(95% CI)
**All**	3265	369162	8.84	4806	835106	5.75	1.88(1.76, 2.01)***
**Age, year**							
≦49	25	89256	0.28	14	146512	0.10	3.07(1.57, 6.00)**
50–64	216	90550	2.39	218	198255	1.10	3.64(2.16, 6.14)***
65–79	1503	134569	11.17	2323	353833	6.57	2.53(2.18, 2.93)***
≥ 80	1521	54786	27.76	2251	136507	16.49	2.12(1.78, 2.53)***
**Sex**							
Female	1428	150200	9.51	2044	341953	5.98	1.93(1.67, 2.22)***
Male	1837	218961	8.39	2762	493153	5.60	1.95(1.74, 2.17)***
**Comorbidity**^**§**^							
No	468	87174	5.37	603	201382	2.99	3.73(2.89, 4.82)***
Yes	2797	281988	9.92	4203	633724	6.63	1.77(1.64, 1.91)***

PY, person-years; Rate^#^, incidence rate per 1000 person-years

HR, relative hazard ratio

Comorbidity^§^: Patients with any one of the comorbidities (diabetes, hypertension, hyperlipidemia, head injury, depression, stroke, COPD, CAD, CHF, AF, cancer, liver disease, chronic infection/inflammation, autoimmune disease, or malnutrition) were classified as the comorbidity group

**P < .01,

***P < .001

The results of the Cox proportional hazards regression models for analyzing the risk of variables contributing to dementia are shown in Table 3. The HR of dementia was increased 1.10-fold (95% CI = 1.09–1.10) with age (every year). The risks of dementia were higher in patients with comorbidities, namely diabetes (HR = 1.15, 95% CI = 1.05–1.26), hypertension (HR = 1.84, 95% CI = 1.67–2.03), depression (HR = 1.78, 95% CI = 1.39–2.28), stroke (HR = 2.63, 95% CI = 2.36–2.93), COPD (aHR = 2.31, 95% CI = 2.03–2.62), CAD (HR = 1.67, 95% CI = 1.50–1.86), CHF (HR = 2.43, 95% CI = 2.00–2.95), chronic infection/inflammation (HR = 1.26, 95% CI = 1.03–1.55), and malnutrition (HR = 4.06, 95% CI = 2.72–6.07).

**Table 3:** HR of dementia in association with sex, age, and comorbidities in Cox proportional hazards regression models.

Variable	HR	(95% CI)
**AKI**	1.88	(1.76, 2.01)***
**Sex (men vs women)**	1.01	(0.92, 1.11)
**Age, years**	1.10	(1.09, 1.10)***
**Baseline comorbidities (no vs yes)**		
Diabetes	1.15	(1.05, 1.26)**
Hypertension	1.84	(1.67, 2.03)***
Hyperlipidemia	0.98	(0.85, 1.13)
Head injury	0.86	(0.72, 1.04)
Depression	1.78	(1.39, 2.28)***
Stroke	2.63	(2.36, 2.93)***
COPD	2.31	(2.03, 2.62)***
CAD	1.67	(1.50, 1.86)***
CHF	2.43	(2.00, 2.95)***
AF	1.93	(1.59, 2.34)***
Cancer	0.48	(0.41, 1.00)
Liver disease	0.72	(0.64, 1.01)
Chronic infection/inflammation	1.26	(1.03, 1.55)*
Autoimmune disease	1.35	(0.83, 2.20)
Malnutrition	4.06	(2.72, 6.07)***

HR, relative hazard ratio;

*p<0.05,

**p<0.01,

***P < .001

## Discussion

AKI is defined as a rapid decline in glomerular filtration rate (GFR), resulting in the retention of nitrogenous wastes such as creatinine and blood urea nitrogen [17]. The incidence of AKI has been reported to be approximately 5%–7% in hospitalized patients [9, 10, 18], and it may be even higher in critical care patients [19]. In addition to decreased renal blood flow causing a decline in GFR, inflammation also represents a major component in AKI [17], leading to a more extensive phase of injury and remote effect. Increasing evidence has suggested that AKI mediates a systemic response that can lead to multiple end-organ damage including the heart, liver, lungs, and brain [20–24]. The association between AKI and long-term adverse outcomes remains controversial, which is probably due to the reversible nature of clinical AKI. However, patients who survive an episode of AKI have a significantly increased risk of developing advanced CKD [15, 25]. Previous studies have also indicated that patients who recover from AKI have a predisposition to cardiovascular disease, cerebrovascular event and higher mortality [9, 13–16]. The association between AKI and poor long-term outcomes remains under debate. In the current nationwide cohort study, we have demonstrated that hospitalized patients surviving AKI would have higher probability of developing dementia in the long term compared to patients without AKI.

The brain and kidneys share anatomic and hemodynamic similarities. They are both low-resistance end organs exposed to high-volume blood flow, which renders them more susceptible to vascular damage compared with other organs [26]. In cases of AKI, vascular injury can result in endothelial dysfunction, causing loss of vasomotor regulatory ability. Alterations in endothelial permeability, endothelial–leukocyte interactions, and coagulations also play roles in subsequent detrimental impacts [27]. Cerebral small vessels might have a similar pathogenesis of vascular injury to the glomerular vessels found with AKI, with increased vascular permeability and subsequent damage. Marked cellular abnormalities and upregulation of microglia within the central nervous system have also been reported in a rat model of AKI [20]. This supports the hypothesis that the brain might also be affected through remote oxidative stress, synergistic inflammatory cascades, activation of proapoptotic pathways, and differential molecular expression [22, 24, 28]. Both alteration in cerebral blood vessels and neuroinflammation have a profound influence on cognitive function [29, 30].

Although several epidemiological studies have established a clear association between CKD and dementia [31–33], our study is the first epidemiological study suggesting a possible link between AKI and dementia. In a population-based cohort study, Sasaki et al concluded that CKD was strongly associated with the incidence of dementia independent of vascular risk factors [32]. In another population-based cohort study in Taiwan, patients with CKD were shown to be at an elevated risk of dementia after comorbidities and medication were controlled [33]. A rapid decline in renal function was associated with global cognitive decline, probably through the vascular mechanism [31]. In the current study, which was conducted using a large sample in a nationwide population-based cohort, we observed that AKI was associated with dementia. A substantial concern is that the CKD following AKI might partially contribute to the increasing risk of subsequent dementia. In this inpatient cohort, 93% of AKI patients were free of a later CKD diagnosis. The risk of dementia development was still significantly higher in these patients. Although we have controlled age, sex, and numerous comorbidities, it is highly possible that AKI is a clinical predictive marker for other unmeasured risk factors, such as disease severity, the length of hospital stays, medication, or other comorbidities that were not included for analysis in this study. AKI itself might also serve as an individual risk factor.

Despite the controversial classification of dementia on the basis of its underlying neuropathology, cerebrovascular dysfunction is a common feature at a point during the disease process [30]. The vascular risk factors are substantial and remain treatable. Our study demonstrates that in addition to AKI, other vascular risk factors also contribute to dementia development, such as diabetes and hypertension. Other comorbidities that are associated with dementia included depression, stroke, COPD, CAD, CHF, AF, chronic infection/inflammation, and malnutrition.

Several limitations were encountered while conducting this study. First, we used ICD-9-CM codes instead of clinical assessment and laboratory data to identify the diagnosis of AKI. These may lead to less accurate results; It lacked the information about AKI etiology, stage, and recovery status. It would be expected that most AKI is AKI stage 1, which recovers quickly with litter consequences. However, patients who are coded with AKI may be more likely to be in severe stage (stage 2/3). All these factors reflect the disease severity and might subsequently influent the risks of dementia development. Second, pre-existing cognitive impairment, which could not be determined using this database, might contribute to the subsequent dementia. Despite our having controlled numerous factors, residual confounding effects before or after the AKI still exist. We hypothesized that AKI is related to the long-term dementia development. It is possible that patients with AKI carry more vascular or other immeasurable risks even before the AKI event. The rapid curve separation in Fig 1 suggests factors before the AKI episode might also contribute to the dementia development. Furthermore, death could not be handled as a competing risk rather than censored because the mortality data was unavailable in the current study. This could result in over-emphasizing the effect size. Most crucially, evidence derived from a retrospective cohort study is typically lower in statistical quality because of numerous sources of inherent bias, including the classification bias. However, the NHI program has high coverage rate, and medical reimbursement specialists and peer review scrutinized all the insurance claims, making sure the diagnoses and coding of diseases were highly reliable in the NHIRD. The classification bias was also supposed to be non-differential, and should not invalidate our result. The relationship between AKI and dementia must be interpreted very cautiously and we could only conclude that there may be an association between the AKI episode and the future diagnosis of dementia.

## Conclusion

In the current study, we observed a significantly increased risk of dementia in patients with AKI by using a large nationwide population-based cohort. Although the overall incidence for dementia is low (8.84 per 1000 person-years), these data suggest that AKI may serve as a risk factor for long-term functional changes in the brain. Additional clinical studies investigating the related pathways are warranted.

## Supporting information

S1 STROBE Checklist Checklist of items that should be included in reports of observational studies. (DOC)

## Abbreviations

aHR: adjusted hazard ratio
AKI: acute kidney injury
CI: confidence interval
ESRD: end-stage renal disease
ICD-9-CM: International Classification of Diseases, 9th Revision, Clinical Modification
LHID 2000: Longitudinal Health Insurance Database 2000
NHIRD: National Health Insurance Research Database
